# Next-generation sequencing (NGS) analysis and age-based survival comparison among glioblastoma (GBM) patients: a two-center cohort study

**DOI:** 10.1007/s00701-026-06924-1

**Published:** 2026-06-23

**Authors:** Chul Ou Lee, Sun Yong Park, Yejin Yun, Ju Young Choi, Jae-Sung Park, Sin-Soo Jeun, Yeo Song Kim

**Affiliations:** 1 Department of Neurosurgery, College of Medicine, Seoul St. Mary’s Hospital, The Catholic University of Korea, Seoul, Republic of Korea; 2 College of Medicine, The Catholic University of Korea, Seoul, Republic of Korea; 3 Department of Neurosurgery, College of Medicine, Incheon St. Mary’s Hospital, The Catholic University of Korea, Seoul, Republic of Korea; 4 Department of Neurosurgery, GSAM Hospital, 591, Gunpo-ro, Gunpo-si, Gyeonggi-do 15839 Republic of Korea

**Keywords:** Glioblastoma, Next-generation sequencing, Age, Cancer

## Abstract

**Objective:**

Glioblastoma (GBM) incidence increases with age, and its etiologies may differ between young and elderly patients. This study aimed to investigate genetic differences between young and elderly GBM patients using next-generation sequencing (NGS) data.

**Methods:**

We retrospectively analyzed 124 GBM patients (< 65 years, *n* = 60; ≥ 65 years, *n* = 64) who underwent surgery at two institutions between 2017 and 2022. Clinical and NGS molecular data were analyzed, and survival analysis used the Kaplan–Meier method and Cox regression models.

**Results:**

The elderly group (≥ 65 years) had a significantly lower OS of 11.0 months compared to 18.0 months in the younger group (*p* = 0.001). The Stupp protocol, a known prognostic factor, was more frequent in the younger group (*p* = 0.023). Molecular analysis showed that MGMT methylation was more prevalent in elderly patients than in younger patients (*p* = 0.009). NGS genetic results showed that ATRX mutations were more common in younger patients than in elderly patients (*p* = 0.052, Fisher’s exact test) but this was not statistically significant. Trends toward higher MET and CDK6 amplification rates were observed in the elderly group compared to the younger group (*p* = 0.058, Fisher’s exact test), although these did not reach statistical significance. Multivariate analysis confirmed that advanced age (HR = 1.961, *p* = 0.002), MGMT methylation (HR = 0.513, *p* = 0.004), the Stupp protocol (HR = 0.376, *p* < 0.001), and GTR (HR = 0.519, *p* = 0.002) were independent prognostic factors.

**Conclusion:**

MGMT methylation was significantly more prevalent in elderly GBM patients and favorably influenced prognosis. No NGS-derived genetic alterations reached statistical significance between age groups after Fisher’s exact test and multiple testing correction. Larger multicenter studies are needed to validate these exploratory findings.

**Supplementary Information:**

The online version contains supplementary material available at 10.1007/s00701-026-06924-1.

## Introduction

Glioblastoma (GBM) represents the most common and aggressive primary brain tumor in adults, characterized by rapid growth, infiltrative nature, and poor prognosis [26]. Despite advances in surgical techniques, radiation therapy, and chemotherapy, the median overall survival (OS) for GBM patients remains dismally low, typically ranging from 12 to 15 months following diagnosis​​ [12]. Standard treatment protocols, including maximal safe resection followed by concurrent radiotherapy and temozolomide, have marginally improved survival but have not significantly altered the disease's lethal trajectory [3]. The heterogeneity of GBM at the molecular level contributes significantly to its treatment resistance and recurrence [10].

Brain aging and neurodegenerative changes are associated with increased expression of specific genes, which are known to play a role in all cancers, neuroinflammation, and neurodegeneration [9]. Therefore, the incidence of GBM increases with age, peaking between the ages of 70 and 79 [9]. Nevertheless, GBM also occurs in younger populations. This raises the question of whether the etiologies of GBM in younger individuals differ from those in the elderly [6].

South Korea has provided insurance coverage for Next-generation sequencing (NGS) for glioma patients since 2017 [13]. Therefore, most patients who underwent surgery for GBM at our institutions since 2017 have had NGS performed. NGS has revolutionized the molecular characterization of GBM by enabling comprehensive profiling of genetic and epigenetic alterations [17]. Through NGS, we aim to investigate whether there are genetic differences in GBM according to age.

## Methods

### Patient selection

This retrospective study was approved by the Institutional Review Board (IRB) of our institution (OC24WCDI0083). The requirement of informed consent was waived by the IRB due to its retrospective nature. Since 2017, our two institutions have performed NGS on patients with glioma. Between 2017 and August 2022, 96 patients were diagnosed with grade IV glioma at Seoul St. Mary’s Hospital, and 41 patients were diagnosed with grade IV glioma at Incheon St. Mary’s Hospital. According to the 2021 EANO guidelines and WHO CNS classification, 85 patients were diagnosed with GBM, IDH-wildtype at Seoul St. Mary’s Hospital and 39 patients were diagnosed with GBM, IDH-wildtype at Incheon St. Mary’s Hospital [18, 28]. We tracked the prognosis of these patients until April 2024.

### Data collection

Clinical and molecular data were extracted from the electronic medical records (EMR) and NGS reports. The clinical variables included sex, age at diagnosis, and OS. In this study, old age was defined as 65 years or older [9]. We accounted for the treatment variables of the Stupp protocol [26] and gross total resection (GTR). After tumor surgery, brain magnetic resonance imaging (MRI) was conducted within 1–3 days of the initial surgery. GTR was defined as the removal of at least 90% of the tumor intraoperatively, as confirmed on postoperative MRI [2]. We performed molecular analysis and targeted panel-based NGS analysis of tumors extracted from patients. These NGS samples underwent amplicon capture-based library preparation and were sequenced using the Oncomine Comprehensive Assay platform [14].

### Statistical analysis

All statistical analyses were conducted using R version 4.1.0 (R Foundation for Statistical Computing, Vienna, Austria). Descriptive statistics were used to summarize the data. The Chi-square test was applied to compare categorical variables between the age groups. Fisher’s exact test was used for all comparisons of genetic alterations identified by NGS between the two age groups. The Benjamini–Hochberg method was applied to adjust for multiple comparisons across the NGS panel. Survival analysis was conducted using the Kaplan–Meier method. Univariate and multivariate Cox regression analyses were performed to identify factors associated with OS. Hazard ratios (HR) and 95% confidence intervals (CI) were calculated. P values were considered significant at less than 0.05 [20].

## Results

### Baseline characteristics

A total of 124 patients diagnosed with GBM wild type were included in this study, and divided into two cohorts based on age: those under 65 years (*n* = 60) and those 65 years and older (*n* = 64) (Table 1). The mean age for the younger cohort was 52.6 ± 11.0 years, while the mean age for the older cohort was 73.4 ± 6.2 years. The gender distribution was similar across both cohorts, with 29 (48.3%) females and 31 (51.7%) males in the younger group, compared to 32 (50.0%) females and 32 (50.0%) males in the older group (*p* = 0.853). A significantly greater number of patients completed the Stupp protocol in the younger compared to the older age group (younger group = 42 (70.0%) vs. older group = 32 (50.0%), *p* = 0.023). There was a higher prevalence of the MGMT promoter methylation status in the older group, with 34 (53.1%) compared to 18 (30.0%) in the younger group, with statistical significance (*p* = 0.009). OS in the younger age group was significantly longer at 18.0 months compared to the older age group at 11.0 months (*p* = 0.001). A summary of the patient demographics is outlined in Table 1.

**Table 1:** Baseline characteristics of older age and younger age

		Age < 65 (*n* = 60)	Age ≥ 65 (*n* = 64)	*p* value
Sex	Female	29 (48.3%)	32 (50.0%)	0.853
	Male	31 (51.7%)	32 (50.0%)	
Age (years), mean ± SD		52.6 ± 11.0	73.4 ± 6.2	<0.001
Stupp protocol	No	18 (30.0%)	32 (50.0%)	0.023
	Yes	42 (70.0%)	32 (50.0%)	
GTR	No	22 (36.7%)	32 (50.0%)	0.135
	Yes	38 (63.3%)	32 (50.0%)	
MGMT methylation	No	42 (70.0%)	30 (46.9%)	0.009
	Yes	18 (30.0%)	34 (53.1%)	
OS, median		18.0 [12.0; 26.5]	11.0 [5.5; 19.0]	0.001
Status OS	Alive	12 (20.0%)	8 (12.5%)	0.256
	Dead	48 (80.0%)	56 (87.5%)	

*GTR* gross total resection, *MGMT* O6-methylguanine DNA methyltransferase, *OS* overall survival

### Molecular characteristics

Genetic alterations showing trends toward differences between the GBM groups aged 65 and above and those aged below 65 included the ATRX mutation, MET amplification, and CDK6 amplification (Table 2). A complete list of all tested genetic alterations is provided in Supplementary Table 1.

**Table 2:** Comparison of notable NGS results by age (Fisher’s exact test)

		Age < 65 (*n* = 60)	Age ≥ 65 (*n* = 64)	*p* value*	BH adj. p
ATRX mutation	No	56 (93.3%)	64 (100.0%)	0.052*	0.909
	Yes	4 (6.7%)	0 (0.0%)		
MET amplification	No	60 (100.0%)	59 (92.2%)	0.058*	0.909
	Yes	0 (0.0%)	5 (7.8%)		
CDK6 amplification	No	60 (100.0%)	59 (92.2%)	0.058*	0.909
	Yes	0 (0.0%)	5 (7.8%)		

*Fisher’s exact test. None of these differences reached statistical significance after Benjamini-Hochberg correction for multiple testing (119 comparisons). A complete list is provided in Supplementary Table 1

*BH* Benjamini-Hochberg

The ATRX mutation tended to be more frequent in the younger group (6.7%) than in the older group (0%) (*p* = 0.052, Fisher’s exact test). The MET amplification tended to be more frequent in the older group (7.8%) than in the younger cohort (0.0%) (*p* = 0.058, Fisher’s exact test). The CDK6 amplification tended to be more frequent in the older group (7.8%) than in the younger group (0.0%) (*p* = 0.058, Fisher’s exact test). However, none of these differences reached statistical significance after applying the Benjamini–Hochberg correction for multiple testing.

### Overall survival

**Fig. 1:**
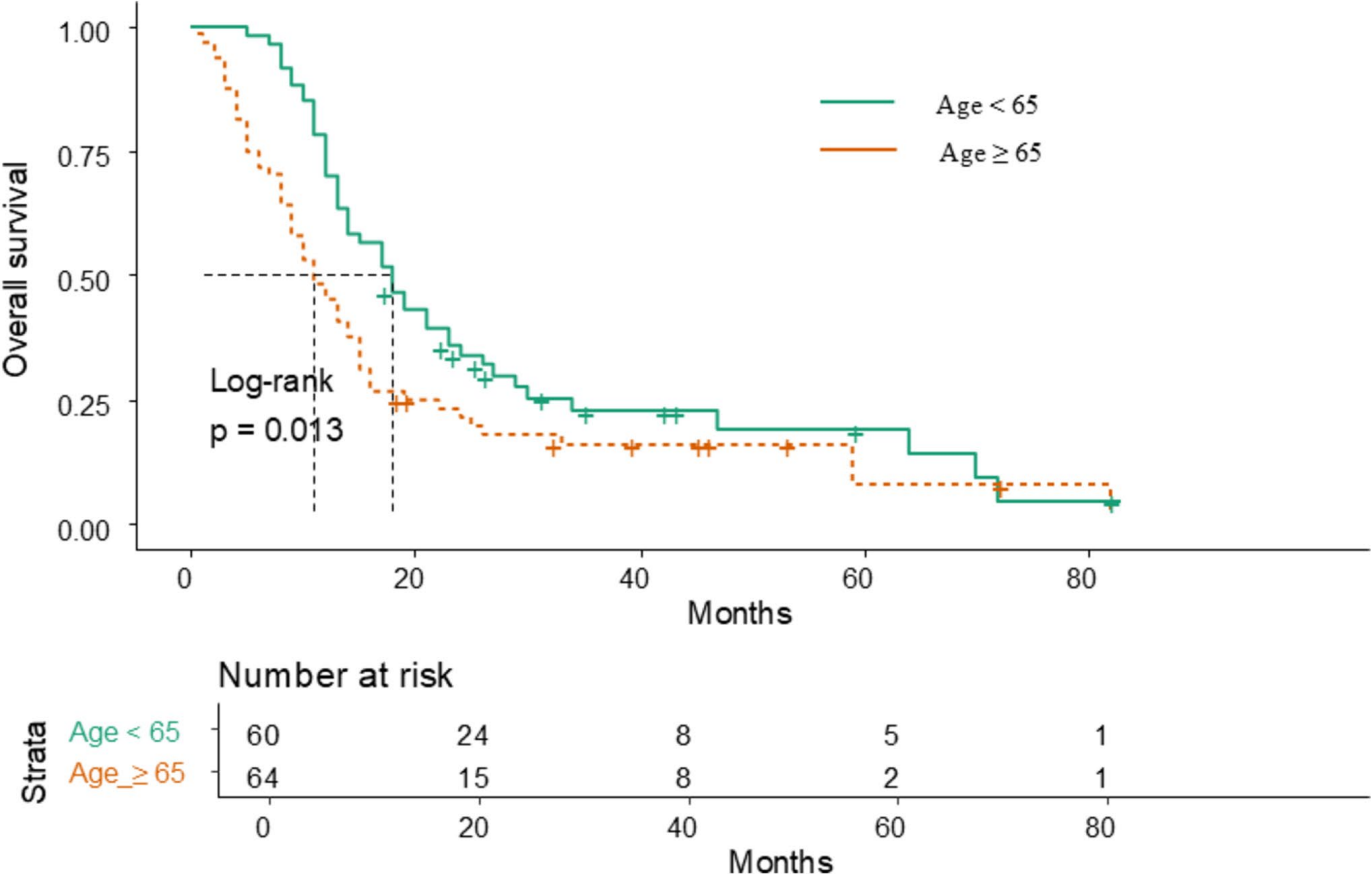
Kaplan–Meier plots representing the probability of overall survival (OS) classified by age

It was confirmed in Kaplan–Meier analysis that individuals under the age of 65 had better outcomes compared to those 65 years and older (Fig. 1). To assess whether factors other than age influence the prognosis, we performed a Cox regression analysis. In the univariate analysis, we investigated sex, age, the presence or absence of the Stupp protocol, GTR rate, MGMT methylation, ATRX mutation, MET amplification, and CDK6 amplification. Univariate Cox regression analysis identified age (HR 1.624, 95% CI 1.103–2.391, *p* = 0.014), MGMT methylation (HR 0.587, 95% CI 0.392–0.879, *p* = 0.010), Stupp protocol (HR 0.348, 95% CI 0.233–0.518, *p* < 0.001) and GTR (HR 0.493, 95% CI 0.334–0.730, *p* < 0.001). The genes that showed differences between old age and young age in the NGS panel were not significant as OS factors: ATRX mutation (HR 0.866, 95% CI 0.274–2.737, *p* = 0.806), MET amplification (HR 1.447, 95% CI 0.529–3.960, *p* = 0.471), CDK6 amplification (HR 1.938, 95% CI 0.785–4.783, *p* = 0.151). Multivariate analysis examined factors that were significant on univariate analysis and NGS genes that were not significant on univariate analysis but varied across age groups. When performing multivariate analysis with only the significant factors, age (HR 1.961, 95% CI 1.275–3.015, *p* = 0.002), MGMT methylation (HR 0.513, 95% CI 0.357–0.805, *p* = 0.004), Stupp protocol (HR 0.376, 95% CI 0.245–0.577, *p* < 0.001), and the GTR rate (HR 0.519, 95% CI 0.341–0.791, *p* = 0.002) were found to be significantly associated with OS (Table 3). Prognostic factors were identified in the elderly patients aged 65 years and over. Only Stupp protocol (HR 0.281, 95% CI 0.148–0.535, *p* < 0.001) and GTR (HR 0.499, 95% CI 0.262–0.951, *p* = 0.035) were significant factors (Table 4).

**Table 3:** Univariate and multivariate Cox regression analyses for overall survival

Variables	Univariate analysis		Multivariate analysis	
	HR [95% CI†]	*p* value	HR [95% CI†]	*p* value
Sex male (vs. female)	1.113 (0.754—1.643)	0.591		
Age ≥ 65 years (vs. < 65 years)	1.624 (1.103—2.391)	0.014	1.961 (1.275—3.015)	0.002
MGMT methylation (vs. non-methylation)	0.587 (0.392—0.879)	0.010	0.513 (0.357—0.805)	0.004
ATRX mutation (vs. no mutation)	0.866 (0.274—2.737)	0.806	0.943 (0.278—3.205)	0.925
MET amplification (vs. no amplification)	1.447 (0.529—3.960)	0.471	1.723 (0.589—5.044)	0.321
CDK6 amplification (vs. no amplification)	1.938 (0.785—4.783)	0.151	0.844 (0.315—2.261)	0.736
Stupp (vs. no Stupp)	0.348 (0.233—0.518)	< 0.001	0.376 (0.245—0.577)	< 0.001
GTR (vs. no GTR)	0.493 (0.334—0.730)	< 0.001	0.519 (0.341—0.791)	0.002

† Confidence interval is 95%. *CI* confidence interval, *HR* hazard ratio

**Table 4:** Univariate and multivariate Cox regression analyses for overall survival in the elderly subgroup (age ≥ 65)

Variables	Univariate analysis		Multivariate analysis	
	HR [95% CI†]	*p* value	HR [95% CI†]	*p* value
Sex male (vs. female)	1.207 (0.709—2.053)	0.488		
MGMT methylation (vs. non-methylation)	0.464 (0.270—0.798)	0.006	0.664 (0.361– 1.221)	0.188
MET amplification (vs. no amplification)	0.991 (0.355—2.761)	0.985	1.450 (0.471–4.469)	0.518
CDK6 amplification (vs. no amplification)	1.411 (0.560—3.556)	0.458	0.973 (0.343–2.758)	0.959
Stupp (vs. no Stupp)	0.216 (0.120—0.386)	< 0.001	0.281 (0.148–0.535)	<0.001
GTR (vs. no GTR)	0.401 (0.228—0.704)	< 0.001	0.499 (0.262–0.951)	0.035

† Confidence interval is 95%. *CI* confidence interval, *HR* hazard ratio

## Discussion

Our study investigated genetic differences and the prognostic impact of genes using molecular analysis and NGS data from elderly and young patient groups. The analysis revealed that GBM patients aged 65 years and older had significantly lower survival rates. MGMT methylation was significantly more prevalent in elderly patients. MET and CDK6 amplifications were more frequently observed in older patients, whereas ATRX mutations were more common in younger patients; however, these differences did not reach statistical significance.

The diagnostic criteria for GBM were redefined in accordance with the 2021 EANO guidelines and WHO CNS classification, which now exclusively define the IDH-wildtype as GBM [28, 31]. Since then, there has been ongoing interest in the differences in disease characteristics based on GBM mutations [7]. Since 2017, the Korean government has subsidized NGS testing for GBM patients, resulting in the majority of GBM patients at our institutions undergoing NGS testing after 2017.

Many previous studies have investigated clinical factors that negatively impact the prognosis of GBM. Among these, numerous studies have reported that older patients tend to have a poorer prognosis compared to younger ones [1, 5–7, 19, 30]. In contrast, some studies suggest that among patients in good postoperative condition who receive standard treatment, the OS between elderly patients aged 65 years and older and those under 65 is similar, suggesting that age alone may not be the only prognostic factor [4]. In these studies, 'elderly' was variably defined as over 60 [25], 65 [1, 5, 7, 19, 30], or 70 years old. However, since 65 was the most used cutoff, we defined elderly patients as those 65 years and older in our study. In our analysis, GBM patients aged 65 years and older had a significantly lower OS of 11.0 months compared to 18.0 months in younger patients (*p* = 0.001). Furthermore, the Stupp protocol and the rate of GTR, which are established prognostic factors in GBM, were higher in younger patients. Therefore, it was necessary to confirm whether these factors contributed to better prognosis in younger patients. Thus, we conducted a multivariate analysis on these factors, and the results confirmed that advanced age was still significantly associated with poor prognosis. Meanwhile, previous studies suggested that genetic mutations could affect the prognosis of GBM, and they classified genes into four groups based on the characteristics of GBM [27]. These studies hypothesized a link between genetic differences and age. Therefore, we hypothesized that genetic differences could account for the prognosis differences between elderly and younger patients.

We reviewed all genes that showed differences in molecular pathology and NGS panels between patients aged 65 years and older and those under 65, and identified MGMT methylation as a statistically significant difference, and observed trends toward differences in ATRX mutations, MET amplification, and CDK6 amplification between the two groups. Numerous studies have reported that the presence of MGMT methylation is significantly associated with better prognosis. MGMT promoter methylation is known to enhance the response to alkylating agents like temozolomide [11, 22]. Patients with MGMT promoter methylation have reduced ability to repair DNA damaged by chemotherapy, resulting in better treatment responses and improved survival rates [22]. However, in our cohort, MGMT methylation was more prevalent in elderly patients. Previous reports have also shown that the methylated patient group, such as ours, tends to be older than the unmethylated group. Therefore, the poor prognosis in the elderly does not appear to be associated with MGMT methylation [8]. In our cohort, the ATRX mutation tended to be more frequent in younger patients, although this did not reach statistical significance (*p* = 0.052). This aligns with a report that found the mean age of patients with ATRX mutation to be 39 years, while the mean age of ATRX wild-type patients was 54 years, indicating that the ATRX mutation is more common in younger patients [24]. Several other reports have also shown the ATRX mutation to be more frequent in younger patients and associated with better prognosis [24, 29, 32]. The CDK6 amplification has been reported as a poor prognostic factor [16]. In our study, CDK6 amplification was more frequently observed in the elderly population, which may have contributed to their poorer prognosis. Meanwhile, amplification and activation of EGFR, PDGFRα, and MET are the top three deregulated RTKs that promote glioma cell proliferation and invasion [23]. Among these, MET amplification is rarely seen in low-grade gliomas but is predominantly found in high-grade gliomas, where it is associated with poor prognosis [15, 21]. In our study, MET amplification was more prevalent in elderly patients, suggesting that it may contribute to the worse prognosis seen in this group.

Both univariate and multivariate analyses confirmed that being aged 65 years and older was associated with reduced survival (HR = 1.961, *p* = 0.002). Furthermore, both analyses identified the Stupp protocol and GTR to be significantly associated with improved survival, underscoring the importance of aggressive treatment strategies. Regarding genetic alterations, MGMT methylation had a significant impact on prognosis, while ATRX mutation, MET amplification, and CDK6 amplification did not. As previously mentioned, some studies have reported that ATRX mutation is associated with a better prognosis, while CDK6 amplification and MET amplification are linked to a worse prognosis. However, in our study, no significant prognostic differences were observed. This may be due to the small sample size of our data.

The relatively small sample size of our cohort (*n* = 124) limits the statistical power to detect meaningful differences in the frequency of individual genetic alterations between age groups, particularly for rare mutations. Additionally, the retrospective two-center design may introduce selection bias. Therefore, our exploratory findings regarding potential age-related trends in MET amplification, CDK6 amplification, and ATRX mutation require validation in larger, prospective multicenter studies. Nonetheless, this study holds value as one of the larger cohorts to investigate NGS profiles in GBM patients according to age.

## Conclusion

Our two-center study investigated NGS profiles stratified by age in GBM patients. Although trends toward higher rates of CDK6 and MET amplification were observed in elderly patients and more frequent ATRX mutations in younger patients, these differences did not reach statistical significance. Furthermore, none of these genes showed a significant impact on survival. However, MGMT methylation was significantly more prevalent in patients aged 65 and older compared to those under 65. Multivariate analysis identified age, MGMT methylation, Stupp protocol, and GTR as independent prognostic factors. Further studies with larger datasets are warranted to determine the significance of age-related genetic differences.

## Supplementary Information

Below is the link to the electronic supplementary material.

ESM 1 Supplementary Material 1 (DOCX 46.4 KB)

## Data Availability

Data cannot be shared publicly due to the violation of patient privacy and the absence of informed consent for data sharing.

## Abbreviations

GBM: Glioblastoma
OS: Overall survival
NGS: Next-generation sequencing
IRB: Institutional Review Board
EMR: Electronic medical records
MRI: Magnetic resonance imaging
GTR: Gross total resection
HR: Hazard ratios
CI: Confidence interval

## Declarations

### Disclaimer

The corresponding author (Yeo Song Kim) had full access to all the data in the study and takes responsibility for the integrity of the data and the accuracy of the data analysis.
