# Supplementary material for: Next-generation sequencing (NGS) analysis and age-based survival comparison among glioblastoma (GBM) patients: a two-center cohort study
Source: Acta Neurochir (Wien). 2026 Jun 23;168(1):166. doi: 10.1007/s00701-026-06924-1 (PMC13415257; doi:10.1007/s00701-026-06924-1)
Supplement: Supplementary file 1 — Supplementary Material 1 (DOCX 46.4 KB) [file 701_2026_6924_MOESM1_ESM.docx]

| **Supplementary Table 1. Complete comparison of next-generation sequencing results by age (Fisher’s exact test)** | | | | | |
| --- | --- | --- | --- | --- | --- |
|  |  | Age < 65  (n = 60) | Age ≥ 65  (n = 64) | Fisher p* | BH adj. p |
| **TP53 mutation** |  | | | | |
|  | No | 40 (66.7%) | 40 (62.5%) | 0.708 | 1.000 |
|  | Yes | 20 (33.3%) | 24 (37.5%) |  |  |
| **TERT mutation** |  |  |  |  |  |
|  | No | 52 (86.7%) | 49 (76.6%) | 0.171 | 0.909 |
|  | Yes | 8 (13.3%) | 1. 23.4%) |  |  |
| - **TERT splicing mutation** | No | 60 (100.0%) | 62 (96.9%) | 0.496 | 0.909 |
|  | Yes | 0 (0.0%) | 2 (3.1%) |  |  |
| - **TERT promoter mutation** | No | 52 (86.7%) | 51 (79.7%) | 0.345 | 0.909 |
|  | Yes | 8 (13.3%) | 13 (20.3%) |  |  |
| **CDKN mutation** |  |  |  |  |  |
|  | No | 47 (78.3%) | 55 (85.9%) | 0.348 | 0.909 |
|  | Yes | 13 (21.7%) | 9 (14.1%) |  |  |
| - **CDKN2A mutation** | No | 47 (78.3%) | 55 (85.9%) | 0.348 | 0.909 |
|  | Yes | 13 (21.7%) | 9 (14.1%) |  |  |
| - **CDKN2B mutation** | No | 50 (83.3%) | 59 (92.2%) | 0.171 | 0.909 |
|  | Yes | 10 (16.7%) | 5 (7.8%) |  |  |
| - **CDKN1B mutation** | No | 59 (98.3%) | 64 (100.0%) | 0.484 | 0.909 |
|  | Yes | 1 (1.7%) | 0 (0.0%) |  |  |
| **PI3K mutation** |  |  |  |  |  |
|  | No | 48 (80.0%) | 54 (84.4%) | 0.639 | 1.000 |
|  | Yes | 12 (20.0%) | 10 (15.6%) |  |  |
| - **PIK3CA mutation** | No | 54 (90.0%) | 56 (87.5%) | 0.779 | 1.000 |
|  | Yes | 6 (10.0%) | 8 (12.5%) |  |  |
| - **PIK3CB mutation** | No | 59 (98.3%) | 64 (100.0%) | 0.484 | 0.909 |
|  | Yes | 1 (1.7%) | 0 (0.0%) |  |  |
| - **PIK3R1 mutation** | No | 58 (96.7%) | 64 (100.0%) | 0.232 | 0.909 |
|  | Yes | 2 (3.3%) | 0 (0.0%) |  |  |
| **FGFR mutation** |  |  |  |  |  |
|  | No | 57 (95.0%) | 63 (98.4%) | 0.353 | 0.909 |
|  | Yes | 3 (5.0%) | 1 (1.6%) |  |  |
| - **FGFR1 mutation** | No | 58 (96.7%) | 63 (98.4%) | 0.610 | 1.000 |
|  | Yes | 2 (3.3%) | 1 (1.6%) |  |  |
| - **FGFR3 mutation** | No | 59 (98.3%) | 64 (100.0%) | 0.484 | 0.909 |
|  | Yes | 1 (1.7%) | 0 (0.0%) |  |  |
| **TSC mutation** |  |  |  |  |  |
|  | No | 57 (95.0%) | 63 (98.4%) | 0.353 | 0.909 |
|  | Yes | 3 (5.0%) | 1 (1.6%) |  |  |
| - **TSC1 mutation** | No | 58 (96.7%) | 64 (100.0%) | 0.232 | 0.909 |
|  | Yes | 2 (3.3%) | 0 (0.0%) |  |  |
| - **TSC2 mutation** | No | 58 (96.7%) | 63 (98.4%) | 0.610 | 1.000 |
|  | Yes | 2 (3.3%) | 1 (1.6%) |  |  |
| **MSH mutation** |  |  |  |  |  |
|  | No | 55 (91.7%) | 63 (98.4%) | 0.106 | 0.909 |
|  | Yes | 5 (8.3%) | 1 (1.6%) |  |  |
| - **MSH2 mutation** | No | 58 (96.7%) | 64 (100.0%) | 0.232 | 0.909 |
|  | Yes | 2 (3.3%) | 0 (0.0%) |  |  |
| - **MSH6 mutation** | No | 57 (95.0%) | 63 (98.4%) | 0.353 | 0.909 |
|  | Yes | 3 (5.0%) | 1 (1.6%) |  |  |
| **NOTCH mutation** |  |  |  |  |  |
|  | No | 57 (95.0%) | 63 (98.4%) | 0.353 | 0.909 |
|  | Yes | 3 (5.0%) | 1 (1.6%) |  |  |
| - **NOTCH1 mutation** | No | 59 (98.3%) | 64 (100.0%) | 0.484 | 0.909 |
|  | Yes | 1 (1.7%) | 0 (0.0%) |  |  |
| - **NOTCH3 mutation** | No | 58 (96.7%) | 64 (100.0%) | 0.232 | 0.909 |
|  | Yes | 2 (3.3%) | 0 (0.0%) |  |  |
| - **NOTCH4 mutation** | No | 60 (100.0%) | 63 (98.4%) | 1.000 | 1.000 |
|  | Yes | 0 (0.0%) | 1 (1.6%) |  |  |
| **SOX mutation** |  |  |  |  |  |
|  | No | 60 (100.0%) | 62 (96.9%) | 0.496 | 0.909 |
|  | Yes | 0 (0.0%) | 2 (3.1%) |  |  |
| - **SOX2 mutation** | No | 60 (100.0%) | 63 (98.4%) | 1.000 | 1.000 |
|  | Yes | 0 (0.0%) | 1 (1.6%) |  |  |
| - **SOX9 mutation** | No | 60 (100.0%) | 63 (98.4%) | 1.000 | 1.000 |
|  | Yes | 0 (0.0%) | 1 (1.6%) |  |  |
| **PTEN mutation** |  |  |  |  |  |
|  | No | 51 (85.0%) | 54 (84.4%) | 1.000 | 1.000 |
|  | Yes | 9 (15.0%) | 10 (15.6%) |  |  |
| **NF1 mutation** |  |  |  |  |  |
|  | No | 48 (80.0%) | 57 (89.1%) | 0.214 | 0.909 |
|  | Yes | 12 (20.0%) | 7 (10.9%) |  |  |
| **EGFR mutation** |  |  |  |  |  |
|  | No | 55 (91.7%) | 55 (85.9%) | 0.399 | 0.909 |
|  | Yes | 5 (8.3%) | 9 (14.1%) |  |  |
| **RB1 mutation** |  |  |  |  |  |
|  | No | 55 (91.7%) | 60 (93.8%) | 0.738 | 1.000 |
|  | Yes | 5 (8.3%) | 4 (6.2%) |  |  |
| **ATRX mutation** |  |  |  |  |  |
|  | No | 56 (93.3%) | 64 (100.0%) | 0.052 | 0.909 |
|  | Yes | 4 (6.7%) | 0 (0.0%) |  |  |
| **SETD2 mutation** |  |  |  |  |  |
|  | No | 58 (96.7%) | 63 (98.4%) | 0.610 | 1.000 |
|  | Yes | 2 (3.3%) | 1 (1.6%) |  |  |
| **PPM1D mutation** |  |  |  |  |  |
|  | No | 59 (98.3%) | 62 (96.9%) | 1.000 | 1.000 |
|  | Yes | 1 (1.7%) | 2 (3.1%) |  |  |
| **EIF1AX mutation** |  |  |  |  |  |
|  | No | 59 (98.3%) | 62 (96.9%) | 1.000 | 1.000 |
|  | Yes | 1 (1.7%) | 2 (3.1%) |  |  |
| **PTPN11 mutation** |  |  |  |  |  |
|  | No | 58 (96.7%) | 63 (98.4%) | 0.610 | 1.000 |
|  | Yes | 2 (3.3%) | 1 (1.6%) |  |  |
| **BRCA2 mutation** |  |  |  |  |  |
|  | No | 58 (96.7%) | 63 (98.4%) | 0.610 | 1.000 |
|  | Yes | 2 (3.3%) | 1 (1.6%) |  |  |
| **MTAP mutation** |  |  |  |  |  |
|  | No | 60 (100.0%) | 62 (96.9%) | 0.496 | 0.909 |
|  | Yes | 0 (0.0%) | 2 (3.1%) |  |  |
| **CREBBP mutation** |  |  |  |  |  |
|  | No | 58 (96.7%) | 64 (100.0%) | 0.232 | 0.909 |
|  | Yes | 2 (3.3%) | 0 (0.0%) |  |  |
| **MLH1 mutation** |  |  |  |  |  |
|  | No | 58 (96.7%) | 64 (100.0%) | 0.232 | 0.909 |
|  | Yes | 2 (3.3%) | 0 (0.0%) |  |  |
| **HLA_A mutation** |  |  |  |  |  |
|  | No | 58 (96.7%) | 64 (100.0%) | 0.232 | 0.909 |
|  | Yes | 2 (3.3%) | 0 (0.0%) |  |  |
| **BRAF mutation** |  |  |  |  |  |
|  | No | 58 (96.7%) | 64 (100.0%) | 0.232 | 0.909 |
|  | Yes | 2 (3.3%) | 0 (0.0%) |  |  |
| **STK11 mutation** |  |  |  |  |  |
|  | No | 60 (100.0%) | 62 (96.9%) | 0.496 | 0.909 |
|  | Yes | 0 (0.0%) | 2 (3.1%) |  |  |
| **PDGFRA mutation** |  |  |  |  |  |
|  | No | 59 (98.3%) | 63 (98.4%) | 1.000 | 1.000 |
|  | Yes | 1 (1.7%) | 1 (1.6%) |  |  |
| **POLE mutation** |  |  |  |  |  |
|  | No | 59 (98.3%) | 63 (98.4%) | 1.000 | 1.000 |
|  | Yes | 1 (1.7%) | 1 (1.6%) |  |  |
| **MAP3K4 mutation** |  |  |  |  |  |
|  | No | 59 (98.3%) | 63 (98.4%) | 1.000 | 1.000 |
|  | Yes | 1 (1.7%) | 1 (1.6%) |  |  |
| **MUTYH mutation** |  |  |  |  |  |
|  | No | 60 (100.0%) | 63 (98.4%) | 1.000 | 1.000 |
|  | Yes | 0 (0.0%) | 1 (1.6%) |  |  |
| **KRAS mutation** |  |  |  |  |  |
|  | No | 60 (100.0%) | 62 (96.9%) | 0.496 | 0.909 |
|  | Yes | 0 (0.0%) | 2 (3.1%) |  |  |
| **FANCA mutation** |  |  |  |  |  |
|  | No | 58 (96.7%) | 64 (100.0%) | 0.232 | 0.909 |
|  | Yes | 2 (3.3%) | 0 (0.0%) |  |  |
| **ARHGAP35 mutation** |  |  |  |  |  |
|  | No | 59 (98.3%) | 64 (100.0%) | 0.484 | 0.909 |
|  | Yes | 1 (1.7%) | 0 (0.0%) |  |  |
| **TET2 mutation** |  |  |  |  |  |
|  | No | 59 (98.3%) | 64 (100.0%) | 0.484 | 0.909 |
|  | Yes | 1 (1.7%) | 0 (0.0%) |  |  |
| **PTCH1 mutation** |  |  |  |  |  |
|  | No | 59 (98.3%) | 64 (100.0%) | 0.484 | 0.909 |
|  | Yes | 1 (1.7%) | 0 (0.0%) |  |  |
| **SMARCB1 mutation** |  |  |  |  |  |
|  | No | 59 (98.3%) | 64 (100.0%) | 0.484 | 0.909 |
|  | Yes | 1 (1.7%) | 0 (0.0%) |  |  |
| **H3_3A mutation** |  |  |  |  |  |
|  | No | 59 (98.3%) | 63 (98.4%) | 1.000 | 1.000 |
|  | Yes | 1 (1.7%) | 1 (1.6%) |  |  |
| **BCOR mutation** |  |  |  |  |  |
|  | No | 59 (98.3%) | 64 (100.0%) | 0.484 | 0.909 |
|  | Yes | 1 (1.7%) | 0 (0.0%) |  |  |
| **ESR1 mutation** |  |  |  |  |  |
|  | No | 60 (100.0%) | 63 (98.4%) | 1.000 | 1.000 |
|  | Yes | 0 (0.0%) | 1 (1.6%) |  |  |
| **PPP2R1A mutation** |  |  |  |  |  |
|  | No | 60 (100.0%) | 63 (98.4%) | 1.000 | 1.000 |
|  | Yes | 0 (0.0%) | 1 (1.6%) |  |  |
| **KMT2D mutation** |  |  |  |  |  |
|  | No | 60 (100.0%) | 63 (98.4%) | 1.000 | 1.000 |
|  | Yes | 0 (0.0%) | 1 (1.6%) |  |  |
| **ZRSR2 mutation** |  |  |  |  |  |
|  | No | 59 (98.3%) | 64 (100.0%) | 0.484 | 0.909 |
|  | Yes | 1 (1.7%) | 0 (0.0%) |  |  |
| **ATM mutation** |  |  |  |  |  |
|  | No | 60 (100.0%) | 63 (98.4%) | 1.000 | 1.000 |
|  | Yes | 0 (0.0%) | 1 (1.6%) |  |  |
| **FNC1 mutation** |  |  |  |  |  |
|  | No | 60 (100.0%) | 63 (98.4%) | 1.000 | 1.000 |
|  | Yes | 0 (0.0%) | 1 (1.6%) |  |  |
| **ASXL2 mutation** |  |  |  |  |  |
|  | No | 59 (98.3%) | 64 (100.0%) | 0.484 | 0.909 |
|  | Yes | 1 (1.7%) | 0 (0.0%) |  |  |
| **LATS1 mutation** |  |  |  |  |  |
|  | No | 59 (98.3%) | 64 (100.0%) | 0.484 | 0.909 |
|  | Yes | 1 (1.7%) | 0 (0.0%) |  |  |
| **CSMD3 mutation** |  |  |  |  |  |
|  | No | 59 (98.3%) | 64 (100.0%) | 0.484 | 0.909 |
|  | Yes | 1 (1.7%) | 0 (0.0%) |  |  |
| **AR mutation** |  |  |  |  |  |
|  | No | 59 (98.3%) | 64 (100.0%) | 0.484 | 0.909 |
|  | Yes | 1 (1.7%) | 0 (0.0%) |  |  |
| **ERCC2 mutation** |  |  |  |  |  |
|  | No | 60 (100.0%) | 63 (98.4%) | 1.000 | 1.000 |
|  | Yes | 0 (0.0%) | 1 (1.6%) |  |  |
| **DICER1 mutation** |  |  |  |  |  |
|  | No | 60 (100.0%) | 63 (98.4%) | 1.000 | 1.000 |
|  | Yes | 0 (0.0%) | 1 (1.6%) |  |  |
| **ARID2 mutation** |  |  |  |  |  |
|  | No | 59 (98.3%) | 64 (100.0%) | 0.484 | 0.909 |
|  | Yes | 1 (1.7%) | 0 (0.0%) |  |  |
| **HDAC9 mutation** |  |  |  |  |  |
|  | No | 59 (98.3%) | 64 (100.0%) | 0.484 | 0.909 |
|  | Yes | 1 (1.7%) | 0 (0.0%) |  |  |
| **FAT1 mutation** |  |  |  |  |  |
|  | No | 59 (98.3%) | 64 (100.0%) | 0.484 | 0.909 |
|  | Yes | 1 (1.7%) | 0 (0.0%) |  |  |
| **DNMT3A mutation** |  |  |  |  |  |
|  | No | 60 (100.0%) | 63 (98.4%) | 1.000 | 1.000 |
|  | Yes | 0 (0.0%) | 1 (1.6%) |  |  |
| **NTRK3 mutation** |  |  |  |  |  |
|  | No | 60 (100.0%) | 63 (98.4%) | 1.000 | 1.000 |
|  | Yes | 0 (0.0%) | 1 (1.6%) |  |  |
| **STAG2 mutation** |  |  |  |  |  |
|  | No | 60 (100.0%) | 63 (98.4%) | 1.000 | 1.000 |
|  | Yes | 0 (0.0%) | 1 (1.6%) |  |  |
| **POLD1 mutation** |  |  |  |  |  |
|  | No | 59 (98.3%) | 64 (100.0%) | 0.484 | 0.909 |
|  | Yes | 1 (1.7%) | 0 (0.0%) |  |  |
| **SMARCA4 mutation** |  |  |  |  |  |
|  | No | 60 (100.0%) | 63 (98.4%) | 1.000 | 1.000 |
|  | Yes | 0 (0.0%) | 1 (1.6%) |  |  |
| **EGFR amplification** |  |  |  |  |  |
|  | No | 47 (78.3%) | 42 (65.6%) | 0.162 | 0.909 |
|  | Yes | 13 (21.7%) | 22 (34.4%) |  |  |
| **CDK4 amplification** |  |  |  |  |  |
|  | No | 48 (80.0%) | 54 (84.4%) | 0.639 | 1.000 |
|  | Yes | 12 (20.0%) | 10 (15.6%) |  |  |
| **PDGFRA amplification** |  |  |  |  |  |
|  | No | 53 (88.3%) | 51 (79.7%) | 0.227 | 0.909 |
|  | Yes | 7 (11.7%) | 13 (20.3%) |  |  |
| **KIT amplification** |  |  |  |  |  |
|  | No | 53 (88.3%) | 55 (85.9%) | 0.792 | 1.000 |
|  | Yes | 7 (11.7%) | 9 (14.1%) |  |  |
| **KDR amplification** |  |  |  |  |  |
|  | No | 56 (93.3%) | 61 (95.3%) | 0.711 | 1.000 |
|  | Yes | 4 (6.7%) | 3 (4.7%) |  |  |
| **MDM4 amplification** |  |  |  |  |  |
|  | No | 56 (93.3%) | 61 (95.3%) | 0.711 | 1.000 |
|  | Yes | 4 (6.7%) | 3 (4.7%) |  |  |
| **MDM2 amplification** |  |  |  |  |  |
|  | No | 58 (96.7%) | 60 (93.8%) | 0.681 | 1.000 |
|  | Yes | 2 (3.3%) | 4 (6.2%) |  |  |
| **PIK3CA amplification** |  |  |  |  |  |
|  | No | 57 (95.0%) | 61 (95.3%) | 1.000 | 1.000 |
|  | Yes | 3 (5.0%) | 3 (4.7%) |  |  |
| **MET amplification** |  |  |  |  |  |
|  | No | 60 (100.0%) | 59 (92.2%) | 0.058 | 0.909 |
|  | Yes | 0 (0.0%) | 5 (7.8%) |  |  |
| **CDK6 amplification** |  |  |  |  |  |
|  | No | 60 (100.0%) | 59 (92.2%) | 0.058 | 0.909 |
|  | Yes | 0 (0.0%) | 5 (7.8%) |  |  |
| **BRAF amplification** |  |  |  |  |  |
|  | No | 60 (100.0%) | 62 (96.9%) | 0.496 | 0.909 |
|  | Yes | 0 (0.0%) | 2 (3.1%) |  |  |
| **CARD11_amplification** |  |  |  |  |  |
|  | No | 60 (100.0%) | 62 (96.9%) | 0.496 | 0.909 |
|  | Yes | 0 (0.0%) | 2 (3.1%) |  |  |
| **RAC1_amplification** |  |  |  |  |  |
|  | No | 60 (100.0%) | 62 (96.9%) | 0.496 | 0.909 |
|  | Yes | 0 (0.0%) | 2 (3.1%) |  |  |
| **RHEB amplification** |  |  |  |  |  |
|  | No | 59 (98.3%) | 63 (98.4%) | 1.000 | 1.000 |
|  | Yes | 1 (1.7%) | 1 (1.6%) |  |  |
| **SMC1A_amplification** |  |  |  |  |  |
|  | No | 59 (98.3%) | 63 (98.4%) | 1.000 | 1.000 |
|  | Yes | 1 (1.7%) | 1 (1.6%) |  |  |
| **AKT3 amplification** |  |  |  |  |  |
|  | No | 59 (98.3%) | 63 (98.4%) | 1.000 | 1.000 |
|  | Yes | 1 (1.7%) | 1 (1.6%) |  |  |
| **AR amplification** |  |  |  |  |  |
|  | No | 59 (98.3%) | 63 (98.4%) | 1.000 | 1.000 |
|  | Yes | 1 (1.7%) | 1 (1.6%) |  |  |
| **CD274 amplification** |  |  |  |  |  |
|  | No | 60 (100.0%) | 63 (98.4%) | 1.000 | 1.000 |
|  | Yes | 0 (0.0%) | 1 (1.6%) |  |  |
| **PCBP1 amplification** |  |  |  |  |  |
|  | No | 60 (100.0%) | 63 (98.4%) | 1.000 | 1.000 |
|  | Yes | 0 (0.0%) | 1 (1.6%) |  |  |
| **SMO amplification** |  |  |  |  |  |
|  | No | 60 (100.0%) | 63 (98.4%) | 1.000 | 1.000 |
|  | Yes | 0 (0.0%) | 1 (1.6%) |  |  |
| **MYCN amplification** |  |  |  |  |  |
|  | No | 60 (100.0%) | 63 (98.4%) | 1.000 | 1.000 |
|  | Yes | 0 (0.0%) | 1 (1.6%) |  |  |
| **NTRK3 amplification** |  |  |  |  |  |
|  | No | 60 (100.0%) | 63 (98.4%) | 1.000 | 1.000 |
|  | Yes | 0 (0.0%) | 1 (1.6%) |  |  |
| **EZHZ amplification** |  |  |  |  |  |
|  | No | 60 (100.0%) | 63 (98.4%) | 1.000 | 1.000 |
|  | Yes | 0 (0.0%) | 1 (1.6%) |  |  |
| **GLI3 amplification** |  |  |  |  |  |
|  | No | 60 (100.0%) | 63 (98.4%) | 1.000 | 1.000 |
|  | Yes | 0 (0.0%) | 1 (1.6%) |  |  |
| **RICTOR amplification** |  |  |  |  |  |
|  | No | 60 (100.0%) | 63 (98.4%) | 1.000 | 1.000 |
|  | Yes | 0 (0.0%) | 1 (1.6%) |  |  |
| **FGFR1 amplification** |  |  |  |  |  |
|  | No | 60 (100.0%) | 63 (98.4%) | 1.000 | 1.000 |
|  | Yes | 0 (0.0%) | 1 (1.6%) |  |  |
| **TERT amplification** |  |  |  |  |  |
|  | No | 59 (98.3%) | 64 (100.0%) | 0.484 | 0.909 |
|  | Yes | 1 (1.7%) | 0 (0.0%) |  |  |
| **ERBB3 amplification** |  |  |  |  |  |
|  | No | 59 (98.3%) | 64 (100.0%) | 0.484 | 0.909 |
|  | Yes | 1 (1.7%) | 0 (0.0%) |  |  |
| **SPOP amplification** |  |  |  |  |  |
|  | No | 59 (98.3%) | 64 (100.0%) | 0.484 | 0.909 |
|  | Yes | 1 (1.7%) | 0 (0.0%) |  |  |
| **ARAF amplification** |  |  |  |  |  |
|  | No | 59 (98.3%) | 64 (100.0%) | 0.484 | 0.909 |
|  | Yes | 1 (1.7%) | 0 (0.0%) |  |  |
| **PIK3C2B amplification** |  |  |  |  |  |
|  | No | 59 (98.3%) | 64 (100.0%) | 0.484 | 0.909 |
|  | Yes | 1 (1.7%) | 0 (0.0%) |  |  |
| **RPS6KB1 amplification** |  |  |  |  |  |
|  | No | 59 (98.3%) | 64 (100.0%) | 0.484 | 0.909 |
|  | Yes | 1 (1.7%) | 0 (0.0%) |  |  |
| **EGFR fusion** |  |  |  |  |  |
|  | No | 56 (93.3%) | 55 (85.9%) | 0.244 | 0.909 |
|  | Yes | 4 (6.7%) | 9 (14.1%) |  |  |
| **CAPZA2 fusion** |  |  |  |  |  |
|  | No | 60 (100.0%) | 62 (96.9%) | 0.496 | 0.909 |
|  | Yes | 0 (0.0%) | 2 (3.1%) |  |  |
| **MET fusion** |  |  |  |  |  |
|  | No | 60 (100.0%) | 62 (96.9%) | 0.496 | 0.909 |
|  | Yes | 0 (0.0%) | 2 (3.1%) |  |  |
| **TACC3 fusion** |  |  |  |  |  |
|  | No | 58 (96.7%) | 64 (100.0%) | 0.232 | 0.909 |
|  | Yes | 2 (3.3%) | 0 (0.0%) |  |  |
| **FGFR3 fusion** |  |  |  |  |  |
|  | No | 58 (96.7%) | 64 (100.0%) | 0.232 | 0.909 |
|  | Yes | 2 (3.3%) | 0 (0.0%) |  |  |
| **PTPRZ1 fusion** |  |  |  |  |  |
|  | No | 59 (98.3%) | 63 (98.4%) | 1.000 | 1.000 |
|  | Yes | 1 (1.7%) | 1 (1.6%) |  |  |
| **KANK1 fusion** |  |  |  |  |  |
|  | No | 59 (98.3%) | 64 (100.0%) | 0.484 | 0.909 |
|  | Yes | 1 (1.7%) | 0 (0.0%) |  |  |
| **NTRK2 fusion** |  |  |  |  |  |
|  | No | 59 (98.3%) | 64 (100.0%) | 0.484 | 0.909 |
|  | Yes | 1 (1.7%) | 0 (0.0%) |  |  |
| **NTRK3 fusion** |  |  |  |  |  |
|  | No | 60 (100.0%) | 63 (98.4%) | 1.000 | 1.000 |
|  | Yes | 0 (0.0%) | 1 (1.6%) |  |  |
| **AKAP13 fusion** |  |  |  |  |  |
|  | No | 60 (100.0%) | 63 (98.4%) | 1.000 | 1.000 |
|  | Yes | 0 (0.0%) | 1 (1.6%) |  |  |
| **PTPR2 fusion** |  |  |  |  |  |
|  | No | 60 (100.0%) | 63 (98.4%) | 1.000 | 1.000 |
|  | Yes | 0 (0.0%) | 1 (1.6%) |  |  |

*Fisher’s exact test was used for all comparisons. BH adj. p = Benjamini-Hochberg adjusted p-value correcting for 119 comparisons. None of the comparisons reached statistical significance after multiple testing correction.

*Abbreviations:* BH, Benjamini-Hochberg
